# Combinations of physical activity, sedentary time, and sleep duration and their associations with depressive symptoms and other mental health problems in children and adolescents: a systematic review

**DOI:** 10.1186/s12966-020-00976-x

**Published:** 2020-06-05

**Authors:** Hugues Sampasa-Kanyinga, Ian Colman, Gary S. Goldfield, Ian Janssen, JianLi Wang, Irina Podinic, Mark S. Tremblay, Travis J. Saunders, Margaret Sampson, Jean-Philippe Chaput

**Affiliations:** 1 School of Epidemiology and Public Health, University of Ottawa, 600 Peter Morand Crescent, Ottawa, Ontario K1G 5Z3 Canada; 2 Healthy Active Living and Obesity Research Group, Children’s Hospital of Eastern Ontario Research Institute, Ottawa, Ontario Canada; 3 Centre for Fertility and Health, Norwegian Institute of Public Health, Oslo, Norway; 4 School of Kinesiology and Health Studies, Queen’s University, Kingston, Ontario Canada; 5 University of Ottawa Institute of Mental Health Research, Ottawa, Ontario Canada; 6 Department of Applied Human Sciences, University of Prince Edward Island, Charlottetown, Prince Edward Island Canada

**Keywords:** Exercise, Recreational screen time, Sleep, Depression, Mental health, Youth

## Abstract

**Background:**

For optimal health benefits, the Canadian 24-Hour Movement Guidelines for Children and Youth (aged 5–17 years) recommend an achievement of high levels of physical activity (≥60 min of moderate-to-vigorous physical activity), low levels of sedentary behaviour (≤2 h of recreational screen time), and sufficient sleep (9–11 h for children or 8–10 h for adolescents) each day. The objective of this systematic review was to examine how combinations of physical activity, sedentary time, and sleep duration relate to depressive symptoms and other mental health indicators among children and adolescents.

**Methods:**

Literature was obtained through searching Medline, EMBASE, PsycINFO, and SportDiscus up to September 30, 2019. Peer-reviewed studies published in English or French were included if they met the following criteria: population (apparently healthy children and adolescents with a mean age of 5–17 years), intervention/exposure (combinations of physical activity, sedentary time, and sleep duration), and outcomes (depressive symptoms and other mental health indicators). A risk of bias assessment was completed for all included studies using the methods described in the Cochrane Handbook. The Grading of Recommendations Assessment, Development and Evaluation (GRADE) framework was used to assess the quality of evidence for each health indicator. Narrative syntheses were employed to describe the results due to high levels of heterogeneity across studies.

**Results:**

A total of 13 cross-sectional studies comprised in 10 papers met inclusion criteria. Data across studies involved 115,540 children and adolescents from 12 countries. Overall, the findings indicated favourable associations between meeting all 3 recommendations and better mental health indicators among children and adolescents when compared with meeting none of the recommendations. There was evidence of a dose-response gradient between an increasing number of recommendations met and better mental health indicators. Meeting the screen time and sleep duration recommendations appeared to be associated with more mental health benefits than meeting the physical activity recommendation. The quality of evidence reviewed was “very low” according to GRADE.

**Conclusions:**

The findings indicate favourable associations between meeting all 3 movement behaviour recommendations in the 24-h guidelines and better mental health indicators among children and adolescents. There is a clear need for high-quality studies that use robust measures of all movement behaviours and validated measures of mental health to increase our understanding in this topic area.

## Background

Depression is a serious public health issue worldwide [1]. It affects more than 300 million people of all ages around the world [2], with a low prevalence in children (less than 1%), and then increases significantly throughout adolescence [3]. Depression is one of the most common major psychiatric disorders that frequently begins during adolescence [4, 5]. However, its negative impacts can extend into adulthood [6, 7]. Several longitudinal studies demonstrated that experiencing depression in early life is associated with a wide range of negative outcomes in adulthood, such as substance use, violent behaviour, and criminal outcomes [8–10]. Depression is the second leading cause of years lived with disability and a leading cause of disability-adjusted life years [11]. It is therefore important to identify modifiable factors that could prevent or alleviate depressive symptoms among children and adolescents.

Achieving high levels of physical activity, low levels of sedentary behaviour, and getting sufficient sleep have been individually associated with better mental health among children and adolescents [12–14]. However, the fact that physical activity, sedentary time, and sleep have been considered separately from each other is concerning, because research has shown that these three behaviours are codependent and should be considered simultaneously [15, 16]. Consequently, the Canadian 24-Hour Movement Guidelines for Children and Youth (aged 5 to 17 years) have been released in 2016 and recommend ≥60 min/day of moderate-to-vigorous physical activity (MVPA), ≤2 h/day of recreational screen time, and 9 to 11 h of sleep per day for 5- to 13-year-olds or 8 to 10 h of sleep per day for 14- to 17-year-olds to support healthy development [17]. However, the prevalence of children and adolescents from different countries around the world who meet all three recommendations contained within the guidelines varies between 3 to 10% [18–23]. Such low prevalence is concerning, so gaining a better understanding of how it relates to mental health is important to study from a public health perspective.

Previous studies looking at the 24-h movement guidelines and health indicators have primarily focused on examining the associations between the combinations of physical activity, sedentary time, and sleep duration with physical health outcomes [12–14]. However, little is known about the extent of the gaps in the current literature regarding different combinations of physical activity, sedentary time, and sleep duration in relation to mental health indicators. It is also unclear whether some combinations of physical activity, sedentary time, and sleep duration could be more beneficial than others. Understanding which combinations are more strongly associated with better mental health is important to help design evidence-informed interventions aimed at improving the health of children and adolescents. This is particularly important given the developmental origins of depression and other mental health conditions begin in childhood and track into adulthood [24]. The systematic review by Saunders et al. [25] on the associations between different combinations of physical activity, sedentary time, and sleep duration with health indicators in children and adolescents is 5 years old and only included studies with objectively-measured physical activity. With the release of movement behaviour guidelines in 2016 [17], there has been a substantial increase in the number of studies investigating indicators of mental health and associations with specific combinations of movement behaviours. Therefore, it is necessary to have a better and current understanding of the combined influence of movement behaviours on mental health indicators. This knowledge will also help to inform and refine public health guidelines.

The purpose of this systematic review was to examine how combinations of physical activity, sedentary time, and sleep duration relate to depressive symptoms in children and adolescents, while secondary aims examined associations with a broader spectrum of mental health indicators. It was hypothesized that meeting all three movement behaviour recommendations would be associated with less depressive symptoms and better overall mental health compared to meeting two, one, or none of the recommendations.

## Materials and methods

### Protocol and registration

This review was registered a priori with the International Prospective Register of Ongoing Systematic Reviews (PROSPERO; submitted 25/11/2019, registration pending), and was conducted following the Preferred Reporting Items for Systematic Reviews and Meta-Analyses (PRISMA) statement for reporting systematic reviews and meta-analyses [26].

### Eligibility criteria

The population, interventions, comparisons, outcomes, and study design (PICOS) framework [27] was followed to identify key study concepts in the research question a priori, and to facilitate the search process.

### Population

Studies of apparently healthy school-aged children and adolescents (aged 5–17 years) were eligible. The mean age of eligible studies had to fall within the bracket of 5 to 17 years, regardless the study sample age range. For example, if the study sample ranged from 11 to 20 years and the mean age was equal to 15 years, the study was included. Studies were excluded if they reported on a clinical sample (e.g., populations exclusively composed of adolescents with depressive symptoms) or if the behaviour (i.e., physical activity, sedentary time, and/or sleep duration) was not measured during the 5–17-year-old age boundaries for at least one time point.

### Intervention (exposure)

Studies were included if they reported *all three* movement behaviours (i.e., physical activity, sedentary time, and sleep duration). Studies were included if they used (1) objective (actigraphy, accelerometry, heart rate monitors, pedometers, arm bands) or subjective (self/proxy-report) measures of physical activity; (2) objective (actigraphy, accelerometry, inclinometer) or subjective (self/proxy-report) measures of sedentary time; and (3) objective (polysomnography, actigraphy, accelerometry) or subjective (self-report, proxy-report) measures of sleep duration.

There are a few specifications worth mentioning here. Objective measures of physical activity can differentiate light-intensity physical activity (LPA) from MVPA, whereas subjective measures capture most often MVPA. For the sedentary component, objective measures report sedentary time, while subjective measures generally report screen time. Sedentary time is defined as the time spent for any duration (e.g., minutes per day) or in any context (e.g., at school or home) in sedentary behaviours, and a sedentary behaviour is any waking behaviour characterized by an energy expenditure ≤1.5 metabolic equivalents (METs), while in a sitting, reclining or lying posture [28, 29]. Screen time refers to the time spent on screen-based behaviours. These behaviours can be performed while being sedentary or physically active [29]. Recreational screen time is the time spent in screen behaviours that are not related to school or work [17].

### Comparison

Various levels and combinations of physical activity, sedentary time, and sleep duration. However, a comparator or control group was not required for inclusion.

### Outcomes

Depressive symptoms represented our primary outcome (indicator) measure. Secondary outcomes (indicators) included other negative (e.g., anxiety, psychological distress, suicidal behaviour) and positive aspects (e.g., flourishing, pro-social behaviour) of mental health, substance use, behavioural problems or disorders (e.g., aggression, child behavioural disorder, child development disorder), and quality of life/well-being.

### Study design

There was no restriction on the types of study designs eligible for inclusion. Only published or in press peer-reviewed articles were included. We excluded case studies and grey literature (e.g., book chapters, dissertations, conference abstracts). For longitudinal studies, any follow-up length was allowed as long as the exposure was measured before follow-up at least once during the identified age range (5–17 years). Follow-up measures of mental health outcomes could occur above this age.

### Information sources and search strategy

The electronic search strategy was created by a research librarian with expertise in systematic review. The following databases were searched: MEDLINE (1946 to September 30, 2019), EMBASE (1947 to September 30, 2019), PsycINFO (1987 to September 29, 2019) using the Ovid interface and SPORTDiscus (from inception to September 29, 2019) using the EBSCO platform. The searches looked for journal articles reporting on sleep, sedentary time and physical activity using previously developed and validated searches [12–14, 30]. Searches did not include terms pertaining to outcomes as this review examines outcomes that may be indexed or categorized in several different manners, and including outcome keywords in our search may lead to exclusion of studies that would meet eligibility criteria otherwise. There was no restriction by type of statistical analyses used. Searches were limited to articles published in English or French. The search strategies are presented in Additional file 1.

### Study selection

After duplicate records were removed online, records retrieved by the electronic search were downloaded and imported into the Reference Manager Software (Thompson Reuters, San Francisco, California, USA) for additional removal of duplicate references. Titles and abstracts of potentially relevant articles were imported to Covidence (a secure, internet-based software; Evidence Partners, Ottawa, Ontario, Canada) where two reviewers screened them independently. Exclusion by both reviewers was required for a study to be excluded at level 1 (title and abstract) screening. At level 2, two independent reviewers performed full-text review of potentially eligible articles. Consensus was required for articles to be included; discrepancies between reviewers were resolved by discussion between them or with a third reviewer, if needed. Reference lists of included articles and relevant reviews were also checked for additional relevant studies.

### Data extraction

Data extraction forms were created by the study coordinators, reviewed by study collaborators, and pilot tested by all reviewers. One reviewer completed the data extraction electronically in Microsoft Excel. A second reviewer independently extracted data from eligible articles and entered this information into the extraction form. Forms were compared afterwards, and discrepancies were resolved by consensus. Reviewers were not blinded to the authors or journals when extracting data. Results were extracted from the most fully adjusted models for studies that reported findings from multiple models.

### Data items

Important study characteristics (e.g., publication year, study design, country, sample size, age and sex of participants, measure of physical activity, sedentary time, sleep, and mental health indicators, results, and confounders) were extracted.

### Risk of bias and study quality assessment

A risk of bias assessment was completed for each outcome within each study using the methods described in the Cochrane Handbook [31]. Following the recommendations for observational studies, the risk of bias assessment identified methodological features of each study that could affect confidence in the overall estimate of effect for an outcome. More specifically, we assessed the risk of selection bias, performance bias, selective reporting bias, detection bias, attrition bias, and potential confounding [32]. Both exposures (i.e. physical activity, screen time, and sleep duration) and outcomes (i.e. depressive symptoms and other mental health indicators) were considered in the assessment of bias related to measurement. When the only source of bias was performance bias due to selection bias due to convenience sampling, this was not considered “serious risk of bias”. Risk of bias was assessed by one reviewer for all included articles, and then verified by another reviewer.

The overall quality of research evidence for mental health indicators was assessed using the Grading of Recommendations Assessment, Development and Evaluation (GRADE) framework [32]. The GRADE framework categorizes the quality of evidence into four groups (“high”, “moderate”, “low”, and “very low”). Evidence quality ratings start at high for randomized studies and at low for all other experimental and observational studies. The quality of evidence can be downgraded if there are limitations across studies in any of the five criteria, including risk of bias, imprecision, inconsistency, indirectness, and publication bias [32]. If there is no cause to downgrade, the quality of evidence can be upgraded if there is a large effect size, there is a dose-response gradient, or if all plausible confounders would decrease an apparent treatment effect. The quality of evidence assessment was evaluated by one reviewer and verified by the larger review team. Disagreements were resolved by discussion among the team members, if needed.

### Data synthesis

Meta-analyses were planned if data were sufficiently homogenous with regards to methodological, statistical, and clinical characteristics. However, a meta-analysis was not possible due to heterogeneous data for the above characteristics. Therefore, a narrative synthesis was used to describe each health indicator.

## Results

### Description of studies

**Fig. 1:**
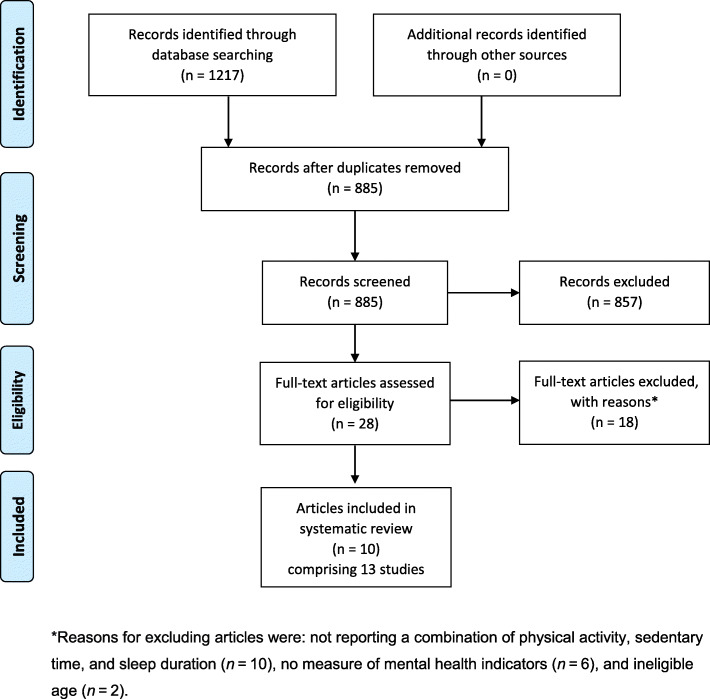
PRISMA flow diagram for the identification, screening, eligibility, and inclusion of studies

A PRISMA flowchart summarizing the article selection process is displayed in Fig. 1. A total of 1217 records were identified during the electronic database search. Of these records, 677 were identified in Medline, 442 in EMBASE, 73 in SportDiscus, and 25 in PsycINFO. After duplicates were removed, a total of 885 records remained. After titles and abstracts were screened, 28 full-text articles were obtained for further review and 10 articles met the inclusion criteria (7 unique samples). Reasons for excluding articles were: not reporting a combination of physical activity, sedentary time, and sleep duration (*n* = 10), no measure of mental health indicators (*n* = 6), and ineligible age (*n* = 2). Some studies were excluded for multiple reasons.

Characteristics of the 10 included articles are summarized in Table 1. Data across studies involved 115,540 children and adolescents from 12 countries. All studies were cross-sectional, and were published in 2016 or later, with participants’ age ranging from 6 to 20 years. The 10 articles comprised 13 studies because some papers had more than one mental health outcome. Of those, 3 reported on depressive symptoms [18, 19, 39], 3 on social and emotional health [33, 34, 37], 2 on health-related quality of life [35, 38], 1 on anxiety [39], 1 on substance use (including cigarette smoking, alcohol consumption, and cannabis use) [18], 1 on impulsivity [36], 1 on cognitive functions [20], and 1 on life satisfaction and prosocial behaviour [37]. Three studies reported on more than one mental health indicator [18, 37, 39]. The included articles used various statistical analyses (Table 1). Three papers used logistic regression analyses [18, 19, 39], 2 papers used compositional data analysis [34, 35], 2 papers used mixed linear models [20, 38], 2 other papers used standard linear regression [33, 37], and 1 paper used structural equation modelling [36].

**Table 1:** Characteristics of included studies

Authors and Study design	Year	Location	Age range (years)	N and statistical analysis	PA measure	SED measure	Sleep measure	Mental health outcome	Main Findings
Carson et al. [33] Cross-sectional	2017	Canada	6–17	4157 (1239 fasting subsample) Linear regression	Accelerometer (Actical, Respironics) MVPA	Questionnaire (Parents/guardians for those aged 6 to 11 years and self-reported by participants aged 12 to 17 years). Screen time	Questionnaire (Parents/guardians for those aged 6 to 11 years and self-reported by participants aged 12 to 17 years). Estimated sleep duration in a 24-h period.	Social and emotional health (Behavioral strengths and difficulties scores)	There was a dose–response pattern between the number of recommendations achieved and social and emotional health. Compared to meeting all three recommendations, meeting none, one, and two recommendations were associated with a higher behavioral strengths and difficulties score in a gradient pattern. Unstandardized beta coefficients and their 95% confidence intervals in parenthesis for meeting 3 > 2 > 1 > 0 recommendations were: 0 (reference group), 0.12 (0.01, 0.24), 0.23 (0.10, 0.37), and 0.34 (0.18, 0.50).
Carson et al. [34] Cross-sectional	2016	Canada	6–17	4169 Compositional data analysis	Accelerometer (Actical, Respironics) LPA and MVPA	Accelerometer (Actical, Respironics) Sedentary time	Questionnaire (Parents/guardians for those aged 6 to 11 years and self-reported by participants aged 12 to 17 years). Estimated sleep duration in a 24-h period.	Social and emotional health (Behavioral strengths and difficulties scores)	The composition of movement behaviours was found to be associated with all health indicators. LPA was positively associated with unfavourable behavioural strengths and difficulties scores, whereas sleep was negatively associated with unfavourable behavioural strengths and difficulties scores.
Dumuid et al. [35] Cross-sectional	2018	Australia, Brazil, Canada, China, Colombia, Finland, India, Kenya, Portugal, South Africa, United Kingdom, and United States	9–11	5855 Compositional data analysis	Accelerometer (Actigraph GT3X+) LPA and MVPA	Accelerometry (Actigraph GT3X+) Sedentary time	Accelerometry (Actigraph GT3X+) Nocturnal sleep duration	Health-related quality of life (KIDSCREEN-10)	The relationship between children’s health-related quality of life and their movement behaviors is moderated by their country’s human development index. In the very high human development index strata alone, health related quality of life was significantly related to the movement behavior composition, with moderate-to-vigorous physical activity (relative to remaining behaviors) being positively associated with health-related quality of life.
Guerrero et al. [36] Cross-sectional	2019	United States	9–10	4524 Structural equation modelling	Questionnaire MVPA	Questionnaire Daily average recreational screen time	Questionnaire (Reported by parents using the Parent Sleep Disturbance Scale for Children) Sleep duration on most night	Impulsivity (UPPS-P Impulsive Behavior Scale34, Behavioral Inhibition System (BIS)/ Behavioral Activation System (BAS) Scale, and cash choice delay discounting task)	Adherence to individual movement behavior recommendations as well as combinations of adherence to movement behavior recommendations were associated with each dimension of impulsivity
Janssen et al. [37] Cross-sectional	2017	Canada	10–17	17,000 Linear regression	Questionnaire MVPA	Questionnaire Average daily screen time	Questionnaire Average nightly sleep duration	Emotional problems, Life satisfaction (Cantril ladder), Prosocial behaviour	Achieving any given recommendation had preferable scores for the health outcomes compared with participants who did not meet the recommendations. There was a dose–response pattern between the number of recommendations achieved and the health outcomes. The adjusted mean and standard errors in parenthesis for meeting 3 > 2 > 1 > 0 recommendations were: 0.72 (0.04), 0.48 (0.03), 0.30 (0.04) and 0.08 (0.07) for emotional problems, −0.82 (0.05), − 0.62 (0.05), − 0.43 (0.05) and − 0.29 (0.09) for life satisfaction, and − 0.37 (0.05), − 0.20 (0.05), − 0.02 (0.06) and 0.11 (0.09) for emotional problems. When the number of recommendations achieved was the same, there were no differences in the health outcomes.
Knell et al. [18] Cross-sectional	2019	United States	13–20	59,397 Logistic regression	Questionnaire MVPA	Questionnaire Average daily screen time	Questionnaire Average nightly sleep duration	Depressive symptoms (2-week sadness), cigarette smoking, alcohol consumption, and cannabis use over the past 30 days	Meeting all 3 recommendations was associated with lower odds of depressive symptoms among males and females, alcohol consumption among females, and cannabis use among males compared to meeting none of the recommendations. Meeting the 3 guidelines was associated with greater odds of smoking cigarette among males compared with meeting none of the recommendations.
Pearson et al. [19] Cross-sectional	2019	United Kingdom	14	3899 Logistic regression	Accelerometer (GENEActiv) MVPA	Questionnaire Screen time on typical weekday	Questionnaire Average nightly sleep duration	Depressive symptoms (Mood and Feelings questionnaire (MFQ))	Meeting all 3 recommendations was associated with lower odds of depressive symptoms among both males and females compared with meeting none of the recommendations.
Sampasa-Kanyinga et al. [38] Cross-sectional	2017	Australia, Brazil, Canada, China, Colombia, Finland, India, Kenya, Portugal, South Africa, United Kingdom, and United States	9–11	6106 Linear mixed models	Accelerometer (Actigraph GT3X+) LPA (not included in statistical analyses) and MVPA	Accelerometry (Actigraph GT3X+) Sedentary time	Accelerometry (Actigraph GT3X+) Nocturnal sleep duration	Health-related quality of life (KIDSCREEN-10)	Children meeting the screen time recommendation, the screen time and sleep recommendation, and all three recommendations had significantly better HRQoL than children not meeting any of these guidelines.
Walsh et al. [20] Cross-sectional	2018	United States	9–10	4524 Linear mixed effects models	Questionnaire MVPA	Questionnaire Daily average recreational screen time	Questionnaire (Reported by parents using the Parent Sleep Disturbance Scale for Children) Sleep duration on most night	Global cognition (Youth NIH Toolbox)	There was a positive gradient between global cognition and each additional recommendation met. Meeting the screen + sleep or screen-only recommendations were the strongest predictors of superior cognition compared to not meeting any recommendations.
Zhu et al. [39] Cross-sectional	2019	United States	6–17	20,708 Logistic regression	Questionnaire All intensities	Questionnaire Daily average recreational screen time	Questionnaire Average weeknight sleep duration	Anxiety and depression (parent- reports)	Meeting all three 24-h movement guidelines was associated with lower odds for anxiety and depression among adolescents compared with meeting none of the recommendations.

*PA* physical activity, *SED* sedentary time, *MVPA* moderate-to-vigorous physical activity, *LPA* light-intensity physical activity

Overall, the prevalence of depressive symptoms was 42.7% (unweighted) in a sample of more than 59,000 adolescents in the United States [18], 14.4% in a sample of nearly 4000 adolescents in the United Kingdom [19], and 10.3% in a sample of more than 35,000 children and adolescents from the United States [39].

The prevalence of children and adolescents who met the physical activity recommendation in the included studies ranged from 4.1 to 40.5%, with the majority in the 33.3–40.5% bracket; those who met the screen time recommendation ranged from 8 to 48.2%, with the majority in the 36.5–48.2% bracket; and those who met the sleep duration recommendation ranged from 41.9 to 89.3%, with the majority in the 66.2–89.3% bracket. The proportion of children and adolescents who met any one recommendation in the included studies ranged from 34.9 to 51.1%, those who met any two recommendations ranged from 18.4 to 37.0%, and those who met all three recommendations ranged from 2.6 to 17.1%.

### Measurement of movement behaviours

Physical activity was measured with accelerometers in 5 studies from 3 unique datasets [19, 33–35, 38], and was either self-reported or parent-reported in the other studies [18, 20, 36, 37, 39]. Sedentary time was objectively measured using accelerometers in 3 studies [34, 35, 38], and self-reported in the rest of the studies [18–20, 33, 36, 37, 39]. Sleep duration was objectively measured in 2 studies [35, 38] and either self-reported or parent-reported in the other studies [18–20, 33, 34, 36, 37, 39].

### Depressive symptoms (primary outcome)

Depressive symptoms were reported in 3 studies [18, 19, 39], representing 3 unique datasets, and 84,004 individual participants with age ranging from 6 to 20 years (Table 2). All three studies had a combined measure of meeting all 3 movement behaviour recommendations (i.e., meeting all 3 vs. none). However, only one study examined different combinations of physical activity, sedentary time, and sleep duration (i.e., meeting 3, 2, 1 vs. none) [39]. Two studies stratified their results by sex [18, 19], while the other study stratified their analysis by age group (6–11 years old vs. 12–17 years old) [39]. Both of the studies that conducted sex-specific analyses found that depressive symptoms were associated with lower odds of meeting all 3 guidelines in both male and female adolescents [18, 19]. The study that conducted age-specific analyses found that meeting all three 24-h movement guidelines was associated with lower odds for anxiety and depression among adolescents (i.e. 12–17 years old) compared to meeting none of the recommendations [39]. Meeting all three recommendations was associated with significantly lower odds of depressive symptoms among adolescents than meeting none, one, or any combination of two recommendations among adolescents [39]. However, among children (i.e. 6–11 years old), meeting the screen time recommendation alone, both the screen time and sleep duration recommendations, or both the physical activity and sleep duration recommendations was associated with lower odds of depressive symptoms than meeting none or all three guidelines [39].

**Table 2:** Combinations of physical activity, sedentary time, and sleep duration and their relationships with depressive symptoms

No of studies	Design	Quality assessment					No of participants	Absolute effect	Quality
		Risk of bias	Inconsistency	Indirectness	Imprecision	Other			
3	Cross-sectional study^a^	Serious risk of bias^b^	No serious inconsistency	No serious indirectness	No serious imprecision	None	84,004	**MEETING ALL 3 RECOMMENDATIONS** [18, 19, 39] 3/3 studies found that meeting all 3 recommendations (compared with meeting none) was associated with lower odds of depressive symptoms among male and female adolescents [18, 19] but not children [39]. **DIFFERENT COMBINATIONS OF PHYSICAL ACTIVITY + SEDENTARY BEHAVIOUR + SLEEP** [39] 1/1 study found that meeting all three recommendations was associated with significantly lower odds of depression among adolescents compared with meeting none, one, or any combinations of two recommendations among adolescents. However, among children, meeting the screen time recommendation alone, both the screen time and sleep duration recommendation, or both the physical activity and sleep duration recommendations was associated with lower odds of depression than meeting none or all three guidelines.	VERY LOW

Age ranged between 6 and 20 years, and all data collection was cross-sectional

^a^Includes 3 cross-sectional studies [18, 19, 39]

^b^All studies used a subjective assessment of movement behaviours with no psychometric properties reported, except one study that measured physical activity by accelerometer [19]. Depression was measured differently across studies, with only one study that used a validated instrument [19]; one study [18] used an item that asked students if they felt sad or hopeless almost every day for two weeks or more in a row in the past year; another study [19] used the Mood and Feelings questionnaire (MFQ), a reliable and valid measure of depression in children, and another study [39] used parents’ response (“currently have condition”) to the question asking if they had ever been told by a health care professional that the child had the condition, and whether the child currently has depression. Therefore, the quality of evidence was downgraded from “low” to “very low” due to a serious risk of bias

Following the GRADE protocol, the quality of evidence for the three studies examining the association between the 24-h movement guidelines and depressive symptoms began with a low-quality rating due to their observational design. Given that only one study used an objective measure of physical activity and a reliable and valid measure of depression in children [19], the quality of evidence was downgraded from “low” to “very low” due to a serious risk of bias.

### Other mental health indicators (secondary indicators)

A total of 10 studies examined the association between the 24-h movement guidelines and other mental health indicators, including 1 on anxiety [39], 3 on social and emotional health [33, 34, 37], 2 on health-related quality of life [35, 38], 1 on substance use (including cigarette smoking, alcohol consumption, and cannabis use) [18], 1 on impulsivity [36], 1 on cognitive functions [20], and 1 on life satisfaction and prosocial behaviour [37] (Table 3). All studies were cross-sectional, involving 111,641 participants aged 6 to 20 years from 6 unique datasets. These studies were consistent in showing better mental health indicators in participants who met all three recommendations compared to those who met none of the recommendations. While some studies found that meeting the recommendations for MVPA, screen time, and sleep duration each had a comparable strength of association with mental health indicators [37, 39], three studies found that meeting the screen time recommendation or the screen time + sleep duration recommendations could have more benefits to mental health [20, 36, 38]. Four studies [33–35, 38] from 2 unique datasets used objective measures of physical activity and only 2 studies [35, 38] used objective measures of sedentary time and sleep duration. Therefore, the quality of evidence was downgraded from “low” to “very low” because of a serious risk of bias (i.e., most studies used a subjective assessment of physical activity, screen time, and sleep duration with no psychometric properties reported). Only two studies [33, 37] examined and documented a positive dose-response gradient between the number of recommendations met with mental health indicators (3 > 2 > 1 > 0), indicating that a greater number of recommendations met was associated with a lower risk of mental health problems. However, other studies did not examine a possible dose-response gradient.

**Table 3:** Combinations of physical activity, sedentary time, and sleep duration and their relationships with other mental health indicators

No of studies	Design	Quality assessment					No of participants	Absolute effect	Quality
		Risk of bias	Inconsistency	Indirectness	Imprecision	Other			
10	Cross-sectional study^a^	Serious risk of bias^b^	No serious inconsistency	No serious indirectness	No serious imprecision	None	111,641	**MEETING ALL 3 RECOMMENDATIONS** [18, 20, 33–39] 10/10 studies found that meeting all 3 recommendations was associated with lower odds of mental outcomes among children and adolescents compared with meeting none. **Anxiety** [39] Among children, meeting all three guidelines was associated with lower odds of anxiety compared to meeting the physical activity, and the screen time recommendations. Among adolescents, meeting all three guidelines was associated with lower odds of anxiety compared to meeting none of the recommendations **Social and emotional health** [33, 34, 37] 3/3 studies found that participants achieving all 3 recommendations had preferable scores for the health outcomes compared with participants who did not meet the recommendations. **Health-related quality of life** [35, 38] 1/2 study found that the relationship between children’s health-related quality of life and their movement behaviors is moderated by their country’s human development index [38]. 1/2 study found that meeting all three recommendations was associated with significantly better HRQoL than not meeting any of these guidelines [35]. **Substance use**^**c**^ [18] 1/1 study found that compared with meeting none of the recommendations, meeting all 3 recommendations was associated with lower odds of alcohol consumption among females, and cannabis use among males. Meeting all 3 recommendations was also associated with greater odds of smoking cigarette among males **Impulsivity** [36] 1/1 study found that meeting all 3 recommendations was associated with better impulsivity scores compared with meeting none. **Cognitive functions** [20] 1/1 study found that meeting all three recommendations was associated with superior global cognition compared with meeting none. **Life satisfaction and prosocial behaviour** [37]. 1/1 study found that participants achieving all 3 recommendations had preferable scores for the health outcomes compared with participants who did not meet the recommendations. **DIFFERENT COMBINATIONS OF PHYSICAL ACTIVITY + SEDENTARY TIME + SLEEP** 9/10 studies assessed different combinations between movement recommendations and mental health outcomes [20, 33–39]. 3/10 studies found that meeting the screen time recommendation alone, and both the screen time and sleep duration recommendations, were consistently associated with better cognitive functions, impulsivity scores and HRQoL [20, 36, 38]. 2/10 studies found a dose-response relationship between the number of recommendations met and mental health outcomes [33, 37]. 2/10 studies used compositional data analyses and found that the composition of movement behaviours was associated with social and emotional health [34] and with HRQoL [35]; whereas 8/10 used traditional methods [18–20, 33, 36–39].	VERY LOW

HRQoL: health-related quality of life

Age ranged between 6 and 20 years, and all data collection was cross-sectional

^a^Includes 10 cross-sectional studies [18, 20, 33–39]

^b^Four studies [33–35, 38] from 2 unique datasets used objective measures of physical activity and only 2 studies [35, 38] used objective measures of sedentary time and sleep duration. Therefore, the quality of evidence was downgraded from “low” to “very low” because of a serious risk of bias (i.e., most studies used a subjective assessment of physical activity, screen time, and sleep duration with no psychometric properties reported)

^c^Including cigarette smoking, alcohol consumption, and cannabis use

## Discussion

This systematic review synthesized peer-reviewed evidence from 13 studies examining the associations between combinations of physical activity, sedentary time, and sleep duration with depressive symptoms and other mental health indicators among children and adolescents aged 5–17 years. A total of 115,540 participants from 12 countries were represented in this review. All studies were published in 2016 or later, consistent with the release of the Canadian 24-Hour Movement Guidelines in 2016 [17]. The overall quality of evidence was rated as “very low” according to the GRADE framework [32]. Collectively, the findings indicate favourable associations between meeting all 3 recommendations and better mental health indicators among children and adolescents when compared with meeting none of the recommendations. In these studies, meeting the screen time and sleep duration recommendations seemed to be more strongly associated with mental health than meeting the physical activity recommendation. However, evidence in relation to each specific mental health indicator was limited. This comprehensive assessment of available evidence highlights the need for continued efforts to promote the 24-h movement guidelines for overall mental health benefits in children and adolescents. It also highlights the need for higher quality research using longitudinal and experimental study designs, robust measures of movement behaviours, and validated measures of mental health indicators to increase our understanding in this topic area.

To our knowledge, this is the first time the relationship between 24-h movement guidelines and mental health indicators in children and adolescents has been systematically reviewed since the release of aforementioned guidelines in 2016 [17]. Our findings are also of importance as they address the three important research gaps areas within the context of the *Canadian 24-Hour Movement Guidelines for Children and Youth* that have been previously identified in a systematic review on the health benefits of combined movement behaviours [25]. Indeed, Saunders et al. [25] highlighted first that existing evidence was mainly based on physical health indicators such as obesity, urging the need for mental and social health indicators to be considered in future studies. Second, it was unclear whether meeting a given specific recommendation was associated with more health benefits than meeting the others. Third, researchers have mainly compared meeting all 3 recommendations versus meeting none, ignoring potential influence of intermediate combinations. Unfortunately, to date, only 13 studies have examined mental health indicators in the context of the 24-h movement guidelines among children and adolescents. These findings highlight the slow growth of new research on the 24-h movement guidelines as they relate to mental health indicators in children and adolescents. Overall, our results provide some support that meeting all 3 recommendations is associated with better mental health among children and adolescents when compared with meeting none of the recommendations. Although previous evidence has shown that physical activity may prevent depression and/or alleviate its symptoms in children and adolescents [40–42], our results indicate that meeting the screen time and sleep duration recommendations were more strongly associated with fewer depressive symptoms and positive mental health than meeting the physical activity recommendation. These findings are interesting and warrant further investigations, along with potential mechanisms linking these lifestyle behaviours and mental health.

The mechanisms explaining the association between excessive screen time and mental health problems may be direct or indirect. Direct pathways could be observed through the content watched on screens, disrupted interpersonal relationships, or through direct cognitive effects, creating low emotional stability and impulsivity [43]. Indirect pathways could be observed through certain intermediate factors, such as insufficient sleep, unhealthy eating behaviours, dissatisfaction with body weight, cyberbullying victimization, etc. [44–47]. Moreover, excessive screen time could reduce physical activity time [48], thus limiting potential benefits of physical activity on mental health. Several possible mechanisms could explain the association between short sleep duration and mental health problems among children and adolescents. First, short sleep duration negatively affects levels of neurotransmitters that regulate mood and thinking [49]. It also impairs executive functions [50], including working memory, cognitive flexibility, and inhibitory control [51, 52]. Second, short sleep duration could be associated with heightened stress reactivity, which has been associated with the activation of the hypothalamic-pituitary-adrenal and the sympatho-adrenal-medullary axes, thus increasing the risk of mental health disorders [53]. Finally, short sleep duration can make the maintenance of healthy active living more difficult due to subsequent daytime sleepiness and fatigue [54, 55]. However, the possibility of reverse causation is not excluded, as excessive screen time may be a coping strategy for individuals who are already suffering from mental health problems [43]. It is also possible that mental health problems precede short sleep duration [56], suggesting that individuals with mental health problems could be more likely to sleep less.

In the current review, we found that the majority (80%) of papers used traditional regression models to examine the associations between 24-h movement behaviours (sleep duration, sedentary time, and physical activity) and mental health indicators in children and adolescent. However, research has indicated that movement behaviours are codependent and require analyses that are sensitive to this collinearity [15, 16, 57]. Compositional data analysis has been suggested to represent the most appropriate analytical approach for understanding the association between 24-h movement behaviours and health indicators, because time is finite over a period of 24 h [58]. However, in the current review, only 2 studies used compositional data analyses [34, 35]. Indeed, the use of these novel statistical analyses in the context of 24-h movement behaviours is still in its infancy, but it is growing. Future research including the use of compositional analyses is needed to examine the association between 24-h movement behaviours and mental health indicators among children and adolescents.

To date, evidence addressing the third research gap area identified by Saunders et al. [25] is still limited. Only two studies have examined and documented a dose-response gradient between the number of recommendations met and mental health outcomes [33, 37], where a greater number of recommendations met was associated with a lower risk of mental health problems. These findings suggest that added mental health benefits may be derived from achieving optimal levels of multiple movement behaviours. For example, Janssen et al. [37] provided evidence for a dose-response gradient between the number of movement behaviour recommendations met and mental health indicators, and found that different intermediate combinations of the recommendations had similar influence on mental health indicators while the number of recommendations met was the same. There is a need for more studies to clarify dose-response gradients and relationships (through intervention studies) between 24-h movement behaviours and mental health indicators.

In this review, we focused on MVPA because it is included in the 24-h movement guidelines and it is the most common indicator that could be measured both objectively and subjectively. Although 5 articles used objective measures of physical activity, light physical activity was also included in 2 articles that used compositional data analyses. None of the 10 papers included in the present systematic review have reported on vigorous-intensity physical activity apart from MVPA. Research has shown that different intensities of physical activity could differentially impact mental health outcomes in children and adolescents [59, 60]. For example, Goldfield et al. [61] found a dose-response relationship between intensity of physical activity and psychological distress whereby vigorous physical activity reduces phycological distress in adolescents compared to moderate and light physical activity. Likewise, the current review focussed on sedentary time, particularly total recreational screen time, and did not look at the time spent in specific types of sedentary behaviours (e.g., watching television, using social media, playing games, reading, eating, travelling in a car). Research has shown that types of sedentary behaviour differentially impact upon mental health indicators [62]. Further research that simultaneously measures specific types of sedentary behaviour and different intensities of physical activity is needed to better understand the impacts of different combinations of sedentary behaviour and movement behaviours on mental health among children and youth.

Another measurement issue that is not considered within the context of 24-h movement guidelines relates to different aspects of sleep, such as sleep efficiency, time to fall asleep, number of awakenings, that may be associated with mental health. Indeed, the “sleep component” of the 24-h movement guidelines focuses on sleep duration. However, different aspects of sleep have been associated with mental health problems among adolescents [63, 64]. Future research examining the association between the 24-h movement guidelines and mental health indicators could account for other aspects of sleep health in their analyses to provide more insights into this important relationship.

### Strengths and limitations

This systematic review has several strengths. First, it uses a comprehensive electronic search process that was created by an experienced research librarian, with the inclusion of a broad range of mental health indicators, comprising emotional, psychosocial, and cognitive health. Second, the review used a rigorous methodology following the PRISMA statement for reporting systematic reviews and meta-analyses [26] and GRADE framework [32]. Finally, to our knowledge, this is the first systematic review of the evidence regarding the associations between 24-h movement guidelines and mental health indicators in children and adolescents since the release of the guidelines in 2016 [17].

There are several limitations worth mentioning. First, the quality of evidence assessed using GRADE was of “very low” quality, mainly because of the cross-sectional nature of all included studies, and the use of subjective measures for physical activity, recreational sedentary time, and sleep duration with no psychometric properties reported, raising concerns about possibility of risk of bias. Second, many studies have used a single item measure of mental health problems (e.g., depressive symptoms) which could raise validity and reliability issues. Third, the cross-sectional design of all the included studies precludes temporality and causal relationships between combinations of physical activity, screen time, and sleep duration and mental health indicators. Thus, future studies using longitudinal or experimental designs are needed to confirm cause-and-effect relationships between movement behaviours and mental health indicators among children and adolescents. Fourth, the present systematic review included only articles published in English or French. Therefore, we did not include any studies that may have been published in other languages. Fifth, a meta-analysis was not possible due to heterogenous data on the measurement of physical activity, screen time, sleep duration, and mental health indicators. Future research using standardized methodology is needed to address this limitation. Finally, the strength of our conclusions is limited by the small number of included studies and lack of longitudinal and intervention studies examining the associations between 24-h movement guidelines and mental health indicators in children and adolescents.

### Future directions

Research evidence is just beginning to emerge regarding the relationships between 24-h movement guidelines and mental health outcomes among children and adolescents, and there is a clear need for future studies to use longitudinal designs, robust measures of movement behaviours, and validated items for mental health indicators. Further research using high-quality study designs will be required to better inform the relationships between 24-h movement guidelines and mental health among children and adolescents. It is also important for future studies to examine potential age and sex differences to inform the design of tailored prevention efforts.

## Conclusions

We systematically reviewed studies that looked at combinations of physical activity, sedentary time, and sleep duration with depressive symptoms and other mental health indicators among children and adolescents. Our review provides supporting evidence that adherence to the 24-h movement guidelines for children and adolescents is associated with better mental health status. These findings underscore the need to encourage children and adolescents to meet the 24-h movement guidelines. It is important that all stakeholders including parents, schools, caregivers, health professionals, policymakers, and children and adolescents themselves be informed about the potential benefits of adherence to the 24-h movement guidelines. However, the available evidence is of very low quality (using the GRADE framework), as it relies heavily on cross-sectional studies using self-reported measures of physical activity, screen time, and sleep duration. Higher quality research is desired to better determine whether a dose-response gradient exists between the number of movement behaviour recommendations met and mental health to better support the 24-h guideline paradigm.

## Supplementary information

**Additional file 1:** Database Search Strategy (MEDLINE, Embase, PsycINFO).

**Additional file 2.**

## Data Availability

Not applicable.
