# Supplementary material for: Combinations of physical activity, sedentary time, and sleep duration and their associations with depressive symptoms and other mental health problems in children and adolescents: a systematic review
Source: Int J Behav Nutr Phys Act. 2020 Jun 5;17:72. doi: 10.1186/s12966-020-00976-x (PMC7273653; doi:10.1186/s12966-020-00976-x)
Supplement: Supplementary file 1 — Additional file 1: Database Search Strategy (MEDLINE, Embase, PsycINFO). [file 12966_2020_976_MOESM1_ESM.docx]

**Database Search Strategy (MEDLINE, Embase, PsycINFO)**

Note: Searches were conducted using an Ovid multi-database search and duplicate records were removed online giving preference to MEDLINE, then Embase. Lines 1-30 are optimized for MEDLINE. Lines 31-53 are optimized for Embase and lines 54-82 are optimized for PsycINFO. The next lines isolate the records to the database the search was designed for, combine those sets and then remove duplicate records and finally isolate the records from each database again so each can be downloaded and imported into the citation manager using a database-specific import filter. During duplicate removal, Medline records are kept in preference to Embase records which are kept in preference to PsycINFO records.

1. Physical Activity.mp.

2. exp Exercise/

3. exp Exercise Movement Techniques/

4. exp Exercise Therapy/

5. Physical Exertion/

6. exp "Physical Education and Training"/

7. exp Sports/

8. (sport$ or bicycl$ or swim$ or walk$ or run$ or jog$).tw,kf.

9. (physical$ adj2 activ$).tw,kf.

10. (aerobic adj2 (train$ or active$)).tw,kf.

11. "Play and Playthings"/ and (activ* or outdoor*).tw,kf.

12. ((activ* or outdoor*) adj3 play*).tw,kf.

13. playground*.tw,kf.

14. active.ti. and (space* or behavio?r* or transport* or commut* or neighbo?rhood* or park* or game* or gaming or lifestyle).mp.

15. (active adj3 (space* or behavio?r* or transport* or commut* or neighbo?rhood* or park* or game* or gaming or lifestyle)).tw,kf.

16. or/1-15

17. Sleep.mp.

18. exp *Sleep Apnea Syndromes/ and (apnea or apnoea).ti.

19. 17 not 18

20. Sedentary Lifestyle/

21. bed rest.mp. or (sedentary or inactiv* or sitting or (lack adj2 activity) or (low adj3 energy expend*)).ti,ab,kf.

22. ((chair or stroller or car or automobile* or auto or bus or motor vehicle* or indoor* or in-door* or screen or computer) adj time).ti,ab,kf.

23. Television/ or Computers/ or Video games/ or Software/ or Videodisc Recording/ or Cartoons as Topic/ or Motion Pictures as Topic/ or exp Internet/ or exp Computers, handheld/ or Communications Media/ or Mass Media/

24. (television or tv or screentime or ((watch* or view*) adj2 (dvd* or video*)) or screen media or social media or video gam* or videogam* or computer gam* or electronic gam*).ti,ab,kf.

25. (screen based entertainment or screen-based entertainment or smartphone* or ipad or apps or app or mobile applications).ti,ab,kf.

26. or/20-25

27. 16 and 19 and 26

28. 27 and (child* or adolesc*).mp.

29. limit 28 to (yr="2009 - 2020" and (english or french) and (journal article or published erratum or "retraction of publication"))

30. exp physical activity/ or exp exercise/ or exp kinesiotherapy/ or physical education/ or exp sport/

31. (sport* or bicycl* or swim* or walk* or run* or jog*).tw,kw.

32. (aerobic adj2 (train$ or active$)).tw,kw.

33. Play/ and (activ* or outdoor*).tw,kw.

34. (((activ* or outdoor*) adj3 play*) or playground*).tw,kw.

35. active.ti. and (space* or behavio?r* or transport* or commut* or neighbo?rhood* or park* or game* or gaming or lifestyle).mp.

36. (active adj3 (space* or behavio?r* or transport* or commut* or neighbo?rhood* or park* or game* or gaming or lifestyle)).tw,kw.

37. or/30-36

38. Sleep/

39. Sleep Time/

40. (sleep adj3 duration).tw.

41. exp Sleep Disordered Breathing/ and (apnea or apnoea).ti.

42. (or/38-40) not 41

43. sedentary lifestyle/

44. bed rest.mp. or (sedentary or inactiv* or sitting or (lack adj2 activity) or (low adj3 energy expend*)).ti,ab,kw.

45. ((chair or stroller or car or automobile* or auto or bus or motor vehicle* or indoor* or in-door* or screen or computer) adj time).ti,ab,kw.

46. video game/ or software/ or videorecording/ or movie/ or exp mass communication/ or television viewing/ or television/ or exp computer/ or internet addiction/ or mobile application/ or exp mobile phone/

47. (television or tv or screentime or ((watch* or view*) adj2 (dvd* or video*)) or screen media or social media or video gam* or videogam* or computer gam* or electronic gam*).ti,ab,kw.

48. (screen based entertainment or screen-based entertainment or smartphone* or ipad or apps or app or mobile applications).ti,ab,kw.

49. or/43-48

50. 37 and 42 and 49

51. 50 and (child* or pediatr* or paediatr* or teen* or adolescen*).mp.

52. limit 51 to (embase and (english or french) and yr="2009 -Current")

53. physical activity/ or exp exercise/ or activity level/ or movement therapy/ or dance therapy/ or mind body therapy/ or energy expenditure/ or physical education/ or exp sports/

54. (sport* or bicycl* or swim* or walk* or run* or jog*).tw,id.

55. (physical* adj2 activ*).tw,id.

56. (aerobic adj2 (train* or active*)).tw,id.

57. (childhood play behavior/ or childhood play development/ or games/ or recreation/) and (activ* or outdoor*).tw,id.

58. ((activ* or outdoor*) adj3 play*).tw,id.

59. playgrounds/ or playground*.tw,id.

60. active.ti. and (space* or behavio?r* or transport* or commut* or neighbo?rhood* or park* or game* or gaming or lifestyle).tw,id.

61. (active adj3 (space* or behavio?r* or transport* or commut* or neighbo?rhood* or park* or game* or gaming or lifestyle)).tw,id.

62. or/53-61

63. Sleep/ or Sleep Deprivation/

64. (sleep adj3 duration).tw.

65. *Sleep Apnea/ and (apnea or apnoea).ti.

66. (63 or 64) not 65

67. Sedentary Behavior/

68. bed rest.mp. or (sedentary or inactiv* or sitting or (lack adj2 activity) or (low adj3 energy expend*)).tw.

69. ((chair or stroller or car or automobile* or auto or bus or motor vehicle* or indoor* or in-door* or screen or computer) adj time).tw.

70. Screen Time/ or Television Viewing/ or exp Computers/ or exp Computer usage/ or Computer Games/ or Digital Gaming/ or Internet/ or exp Communications Media/ or Internet Addiction/ or exp Internet Usage/ or exp Mobile Devices/ or Smartphone/ or Mobile Applications/

71. (television or tv or screentime or ((watch* or view*) adj2 (dvd* or video*)) or screen media or social media or video gam* or videogam* or computer gam* or electronic gam*).tw.

72. (screen based entertainment or screen-based entertainment or smartphone* or ipad or apps or app or mobile applications).tw.

73. or/67-72

74. 62 and 66 and 73

75. 74 and (pre-school* or preschool* or early childhood).mp.

76. limit 74 to (140 infancy <2 to 23 mo> or 160 preschool age )

77. 75 or 76

78. limit 77 to (english or french)

79. limit 78 to ("erratum/correction" or journal article)

80. 79 or (78 and retraction.ti.)

81. limit 80 to yr="2009 -Current"

82. 29 use medall

83. 52 use emczd

84. 81 use psyh

85. or/82-84

SportDiscus Search

| **#** | **Query** | **Results** |  |
| --- | --- | --- | --- |
| S6 | S5 AND (child* OR adolescen* ) | 73 |  |
|  | Limiters - Published Date: 20090101-20191231; Language: English, French; Publication Type: Academic Journal; Document Type: Article |  |  |
| S5 | S4 AND (child* OR adolescen* ) | 104 |  |
| S4 | S1 and S2 and S3 | 300 |  |
| S3 | DE "MASS media" or DE "INTERNET" OR DE "ELECTRONIC games" OR DE "INTERNET games" OR DE "MULTIPLAYER games" OR DE "VIDEO games") or DE "SEDENTARY behavior" or DE "SEDENTARY behavior in children" or (Smartphone* or ipad or apps or app or mobile applications) or screen based entertainment or (television N3 time or screentime or screen N3 time or computer N3 time or (watch* N2 dvd or watch N2 video* or view* N2 dvd* or view N2 video*)) or screen media or social media or video gam* or videogam* or computer gam* or electronic gam* OR chair N3 time or stroller N3 time or car N3 time or automobile* N3 time or auto N3 time or motor vehicle* N3 time or bus N3 time or indoor* N3 time or in-door N3 time or computer N3 time OR sitting or ((sedentary or inactiv* or (lack N2 activity))) or ((low N3 energy expend*) or physical* inactiv*) | 40041 |  |
| S2 | sleep | 11712 |  |
| S1 | TI ( exercise or (physical* n2 activ*) OR aerobic N2 train* or aerobic N2 active* OR sport* or outdoor* OR playground or play or playing OR "rough and tumble" or "active recreation*" OR run* OR walk* OR jog* OR bicycl* or biking or cycling or tricylc* or "tummy time" or "floor time" or prone position or crawl* OR swim* or soccer or gymnastic* ) OR SU (exercise or (physical* n2 activ*) OR aerobic N2 train* or aerobic N2 active* OR sport* or outdoor* OR playground or play or playing OR "rough and tumble" or "active recreation*" OR run* OR walk* OR jog* OR bicycl* or biking or cycling or tricylc* OR swim* or soccer or gymnastic* ) | 883908 |  |
