# Supplementary material for: Combinations of physical activity, sedentary time, and sleep duration and their associations with depressive symptoms and other mental health problems in children and adolescents: a systematic review
Source: Int J Behav Nutr Phys Act. 2020 Jun 5;17:72. doi: 10.1186/s12966-020-00976-x (PMC7273653; doi:10.1186/s12966-020-00976-x)
Supplement: Supplementary file 2 — Additional file 2. [file 12966_2020_976_MOESM2_ESM.doc]

| **Section/topic** | **#** | **Checklist item** | **Reported on page #** |  | |
| --- | --- | --- | --- | --- | --- |
| **TITLE** | | |  |  | |
| Title | 1 | THE STUDY IS IDENTIFIED AS A SYSTEMATIC REVIEW. | 1 |  | |
| **ABSTRACT** | | |  |  | 6-8 |
| Structured summary | 2 | ALL REQUIRED INFORMATION IS PROVIDED IN THE ABSTRACT. | 2-3 |  | |
| **INTRODUCTION** | | |  |  | 8-9 |
| Rationale | 3 | THIS INFORMATION IS PROVIDED. | 4-6 |  | |
| Objectives | 4 | STUDY AIM IS STATED. | 6 |  | |
| **METHODS** | | |  |  | 10 |
| Protocol and registration | 5 | INFORMATION REGARDING THE REGISTRATION IS PROVIDED. | 6 |  | |
| Eligibility criteria | 6 | ELIGIBILITY CRITERIA ARE PROVIDED. | 6-8 |  | |
| Information sources | 7 | ALL INFORMATION SOURCES ARE PROVIDED. | 8 |  | |
| Search | 8 | ELECTRONIC SEARCH STRATEGY IS PROVIDED. | 8-9 |  | |
| Study selection | 9 | THE PROCESS FOR SELECTING STUDIES IS STATED. | 9 |  | |
| Data collection process | 10 | METHOD OF DATA EXTRACTION FROM REPORTS IS DESCRIBED. | 9 |  | |
| Data items | 11 | DATA ITEMS ARE LISTED. | 10 |  | |
| Risk of bias in individual studies | 12 | METHODS USED FOR ASSESSING RISK OF BIAS OF INDIVIDUAL STUDIES ARE DESCRIBED. | 10 |  | |
| Summary measures | 13 | EXPLAINED IN DATA SYNTHESIS | 10 |  | |
| Synthesis of results | 14 | EXPLAINED IN DATA SYNTHESIS | 10 |  | |

Page 1 of 2

| **Section/topic** | **#** | **Checklist item** | **Reported on page #** |  | |
| --- | --- | --- | --- | --- | --- |
| Risk of bias across studies | 15 | EXPLAINED IN DATA SYNTHESIS | 10 |  | |
| Additional analyses | 16 | EXPLAINED IN DATA SYNTHESIS | 10 |  | |
| **RESULTS** | | |  |  | 8 |
| Study selection | 17 | STUDY SELECTION RESULTS ARE PRESENTED. | 11 |  | |
| Study characteristics | 18 | STUDY CHARACTERISTICS ARE PRESENTED. | 11-12 |  | |
| Risk of bias within studies | 19 | DATA ON RISK OF BIAS OF EACH STUDY ARE PROVIDED. | 13-14 |  | |
| Results of individual studies | 20 | RESULTS OF INDIVIDUAL STUDIES FOR ALL OUTCOMES CONSIDERED ARE PROVIDED. | 11-15 |  | |
| Synthesis of results | 21 | PROVIDED IN THE TABLES |  |  | |
| Risk of bias across studies | 22 | PROVIDED IN THE TABLES |  |  | |
| Additional analysis | 23 | NOT APPLICABLE (SEE DATA SYNTHESIS) | 10 |  | |
| **DISCUSSION** | | |  |  | 10 |
| Summary of evidence | 24 | THE MAIN FINDINGS ARE SUMMARIZED. | 15 |  | |
| Limitations | 25 | LIMITATIONS ARE DISCUSSED. | 19-20 |  | |
| Conclusions | 26 | CONCLUSIONS ARE PROVIDED. | 21 |  | |
| **FUNDING** | | |  |  | |
| Funding | 27 | FUNDING INFORMATION IS PRESENTED IN THE DECLARATIONS SECTION. | 22 |  | |

*From:*  Moher D, Liberati A, Tetzlaff J, Altman DG, The PRISMA Group (2009). Preferred Reporting Items for Systematic Reviews and Meta-Analyses: The PRISMA Statement. PLoS Med 6(7): e1000097. doi:10.1371/journal.pmed1000097

For more information, visit: **www.prisma-statement.org**.

Page 2 of 2
